# Impact of a physical activity intervention program on cognitive predictors of behaviour among adults at risk of Type 2 diabetes (*ProActive *randomised controlled trial)

**DOI:** 10.1186/1479-5868-6-16

**Published:** 2009-03-17

**Authors:** Wendy Hardeman, Ann Louise Kinmonth, Susan Michie, Stephen Sutton, the ProActive Project Team

**Affiliations:** 1 General Practice and Primary Care Research Unit, Department of Public Health and Primary Care, Institute of Public Health, University of Cambridge, Robinson Way, Cambridge CB2 0SR, UK; 2 Department of Psychology, University College London, 1-19 Torrington Place, London WC1E 7HB, UK

## Abstract

**Background:**

In the *ProActive *Trial an intensive theory-based intervention program was no more effective than theory-based brief advice in increasing objectively measured physical activity among adults at risk of Type 2 diabetes. We aimed to illuminate these findings by assessing whether the intervention program changed cognitions about increasing activity, defined by the Theory of Planned Behaviour, in ways consistent with the theory.

**Methods:**

*N *= 365 sedentary participants aged 30–50 years with a parental history of Type 2 diabetes were randomised to brief advice alone or to brief advice plus the intervention program delivered face-to-face or by telephone. Questionnaires at baseline, 6 and 12 months assessed cognitions about becoming more physically active. Analysis of covariance was used to test intervention impact. Bootstrapping was used to test multiple mediation of intervention impact.

**Results:**

At 6 months, combined intervention groups (face-to-face and telephone) reported that they found increasing activity more enjoyable (affective attitude, *d *= .25), and they perceived more instrumental benefits (e.g., improving health) (*d *= .23) and more control (*d *= .32) over increasing activity than participants receiving brief advice alone. Stronger intentions (*d *= .50) in the intervention groups than the brief advice group at 6 months were partially explained by affective attitude and perceived control. At 12 months, intervention groups perceived more positive instrumental (*d *= .21) and affective benefits (*d *= .29) than brief advice participants. The intervention did not change perceived social pressure to increase activity.

**Conclusion:**

Lack of effect of the intervention program on physical activity over and above brief advice was consistent with limited and mostly small short-term effects on cognitions. Targeting affective benefits (e.g., enjoyment, social interaction) and addressing barriers to physical activity may strengthen intentions, but stronger intentions did not result in more behaviour change. More powerful interventions which induce large changes in TPB cognitions may be needed. Other interventions deserving further evaluation include theory-based brief advice, intensive measurement of physical and psychological factors, and monitoring of physical activity. Future research should consider a wider range of mediators of physical activity change, assess participants' use of self-regulatory strategies taught in the intervention, and conduct experimental studies or statistical modelling prior to trial evaluation. ISRCTN61323766.

## Background

Physical inactivity is a major public health problem [1], but evidence for the effectiveness of interventions targeting individuals is mixed. A recent review of randomised and non-randomised studies concluded that brief advice by primary care practitioners showed promise, but there was uncertainty about the most effective approaches among high-risk groups [2]. Calls have been made for intervention studies with an explicit theory base [3], as they allow investigation of why interventions are effective or not, and provide insight into mechanisms underlying behaviour change. A review of physical activity intervention studies among adults found scarce and inconsistent evidence about specific mediators of behaviour change based on Social Cognitive Theory and the Transtheoretical Model: cognitive and behavioural processes of change, self-efficacy, decisional balance, social support, and enjoyment [4]. Intervention studies based on the Theory of Planned Behaviour (TPB) showed similar inconsistent evidence about mediators of change in physical activity [5-8] and intentions [8-10].

The *ProActive *Trial evaluated the efficacy of a theory-based intervention program aimed at increasing physical activity, over and above a theory-based brief advice leaflet, among sedentary adults with a parental history of Type 2 diabetes. They constitute an at-risk group as family history of diabetes and sedentary lifestyle strongly interact in predicting future diabetes risk [11]. Participants had intensive biochemical, anthropometric, behavioural and psychological assessment at baseline and 12 months, and behavioural and psychological assessment at 6 months. They were randomised to 1) a brief theory-based leaflet with advice to increase physical activity and a persuasive message emphasizing benefits (*brief advice*, comparison), 2) the leaflet plus an intervention program delivered at participants' homes (*face-to-face*), or 3) the leaflet plus the same intervention program delivered by phone (*distance*). The intervention content was informed by the TPB [12], Self-Regulation Theory [13], Relapse Prevention Theory [14] and Operant Theory [15] (see [16]). The program aimed to increase physical activity by targeting cognitive mediators based on the TPB: instrumental attitude (the extent to which performing the behaviour is good or beneficial), affective attitude (the extent to which it is enjoyable), subjective norm (perceived pressure from important others to perform the behaviour), perceived behavioural control (perceived barriers and facilitators), and intention. The development [16] and implementation [17,18] of the intervention, trial protocol [19], and intervention impact on behavioural, biochemical, physiological and quality of life outcomes [20] have been published.

The intervention program was no more efficacious than brief advice in increasing objectively measured physical activity at 12 months, self-reported activity at 6 and 12 months, or 12-months intention to be more physically active [20]. Participants in all three trial arms increased objective physical activity by the equivalent of 20 minutes of brisk walking per day on average over the year. Measurement of cognitive mediators based on the TPB allowed investigation of the effect of the intervention on cognitions and theory-based mechanisms underlying any effect on cognitions. The measures included global evaluations of performing the target behaviour (e.g., being more physically active is good), and belief-based measures (e.g., being more physically active prevents diabetes).

In this paper we investigate the extent to which the intervention program changed the hypothesised cognitive predictors of physical activity. We hypothesised that the combined intervention groups (face-to-face and distance) would report more positive cognitions about becoming more physically active at 6 and 12 months than the brief advice group. Furthermore, we tested whether any effect on cognitions was consistent with pathways specified by the TPB. We hypothesised that any effect on intention would be explained by changes in instrumental attitude, affective attitude, subjective norm or perceived behavioural control over becoming more physically active.

## Methods

### Participants

Participants were included if they were between 30 and 50 years, had a family history of Type 2 diabetes but no known diabetes, and were sedentary as defined by a screening questionnaire covering occupational and leisure activity [21,22]. Exclusion criteria were being unable to walk briskly on the flat without help for 15 minutes, living outside the reach of the measurement centre and intervention facilitators, serious physical or psychiatric illness limiting involvement in the intervention, life issues interfering with the study, known pregnancy before baseline measurement, and planning to move away [19].

### Measures

#### Demographic, cognitive, behavioural and clinical measures

Baseline measures included gender, age at invitation to the trial, social economic status (UK National Statistics Socio-Economic Classification), body mass index (BMI), smoking status, number of generations with family history of diabetes, worry about diabetes, and perceived risk of developing diabetes compared to other people of the same age.

#### Physical activity

The primary outcome was the ratio of daytime energy expenditure to resting energy expenditure (dayPAR), estimated using heart rate monitoring for three days at baseline and at 12-months follow-up [19,23]. The heart rate-energy expenditure relationship was individually calibrated using oxygen uptake (ml O_2_/kg/body weight) by indirect calorimetry during a sub-maximal graded treadmill exercise test. A secondary outcome was self-reported total physical activity (sum of recreational and work-related activity) over the previous year, expressed in MET hours per week. This was assessed by a validated questionnaire [24] at baseline, 6 and 12 months.

#### Cognitive predictors

Participants completed a self-administered questionnaire based on the TPB [12] at baseline, 6 and 12 months (available from the first author). Items were selected on the basis of an elicitation study in a similar target group, who were asked to list beliefs about becoming more physically active [25]. Items were measured on a Likert-type scale ranging from 1 (*strongly disagree*) to 5 (*strongly agree*). They formed measures of overall evaluations (global measures) and of salient beliefs (belief-based measures).

##### Global measures of cognitions

Attitude comprised *instrumental attitude *(measured with two items: 'being more physically active in the next 12 months would be good/harmful for me') and *affective attitude *(two items: 'for me, being more physically active in the next 12 months would be enjoyable/boring'). Confirmatory factor analysis showed a poor fit for a one-factor model for baseline attitude (χ^2^(2) = 9.862, *p *= .007, standardised root mean square residual (SRMR) = .014, goodness-of-fit index (GFI) = .987, comparative fit index (CFI) = .966, root mean square error of approximation (RMSEA) = .104; p-value of close fit = .061). A two-factor model with instrumental and affective attitude showed better fit (χ^2^(1) = 2.564, *p *= .109, SRMR = .006, GFI = .996, CFI = .993, RMSEA = .066; p-value of close fit = .263), and modification indices suggested no further improvements. Standardised factor loadings for instrumental attitude items were .46 (good) and .63 (harmful, scoring reversed), and for the affective attitude items .65 (boring, scoring reversed) and .83 (enjoyable). The correlation between the two attitude factors was .75. Similar results were found at 6 and 12 months, so a two-factor model was adopted. *Subjective norm *was measured with two items: 'most people who are important to me would want me to become more physically active in the next 12 months', 'most people whose views I value would disapprove if I was more physically active in the next 12 months'. Cronbach's alpha was very low (.34), and the latter item was omitted as its formulation was complex and its correlation with intention much lower compared to the first item. *Perceived behavioural control *included two items: 'it would be difficult for me to be more physically active in the next 12 months even if I wanted to', 'I am confident that I could be more physically active in the next 12 months, if I wanted to'. *Behavioural intention *included 'I intend to be more physically active in the next 12 months', 'it is likely that I will be more physically active in the next 12 months'. For each variable the scores for negatively formulated items were reversed and a mean score calculated across items for each participant. Baseline Cronbach's alphas were satisfactory for affective attitude (.70) and intention (.77), and low for instrumental attitude (.45) and perceived behavioural control (.54). Internal consistency of the measures was similar at 6 and 12 months. The measures showed no significant deviations from normality.

##### Belief-based measures of cognitions

According to the TPB, *belief-based attitude *consists of the perceived probability that the behaviour will produce various outcomes (behavioural beliefs; e.g., 'if I was more physically active it is likely that I would reduce my chances of getting diabetes'), multiplied with the evaluation of those outcomes (e.g., 'reducing my risk of diabetes would be a good thing'). We assessed ten beliefs, which were classified as instrumental ('good for you') or affective ('enjoyable') on the basis of an elicitation study [25]. This was validated by ten raters, who showed substantial agreement (multi-rater kappa = .66, *p *< .0005). Eight outcomes and their evaluation were classified as instrumental (belief-based instrumental attitude), and two as affective (belief-based affective attitude). *Belief-based subjective norm *consists of the perceived behavioural expectations of important referent individuals or groups (normative beliefs; e.g., 'my partner would want me to be more physically active'), multiplied with the participants' motivation to comply with them (e.g., 'generally speaking, I want to do what my partner thinks I should do'). We assessed beliefs in relation to the participants' partner, children, family, and friends. *Belief-based perceived behavioural control *consists of the perceived presence of control factors that may facilitate or impede performance of the target behaviour (e.g., 'it is likely that I will have fewer work commitments'), multiplied with the perceived power of each factor (e.g., 'if I had fewer work commitments it would make it easier for me to be more physically active'). We measured four factors: work commitments, lack of interest, having spare time, and re-organising one's life. When such items are used to compute multiplicative composite scores, the correlations between the composite and other variables may vary depending on the scaling system used (unipolar, e.g., 1 to 5, or bipolar, e.g., -2 to 2). We tested the validity of a multiplicative model, examined various a-priori defined scaling systems, and used optimal scaling to inform how to scale the items. Items were scaled unipolar (0 to 4) for behavioural beliefs, normative beliefs, motivation to comply and control beliefs, and bipolar (-2 to 2) for outcome evaluations and power of control beliefs. Scores on outcome evaluations and power of control belief items were reversed if formulated negatively. For each measure, individual belief combinations were multiplied and then summed. Affective attitude showed some deviation from a normal distribution, but the scale was not transformed as the intervention impact analyses are quite robust against violations of normality. Pearson correlations between global and belief-based measures at baseline were .571 (*p *< .0005) for instrumental attitude, .472 (*p *< .0005) for affective attitude, .552 (*p *< .0005) for subjective norm and .375 (*p *< .0005) for perceived behavioural control.

### Procedure

Ethical approval was obtained from the East of England MREC (Eastern MREC 02/5/53). Participants were recruited between March, 2001, and October, 2003 through their parents with Type 2 diabetes, accessed from 20 general practice diabetes registers in the East of England, or directly from a record of a family history of diabetes in the medical notes of seven practices. All participants gave written informed consent. *N *= 365 participants were randomised. This was carried out centrally by the trial statistician, incorporating a partial minimisation procedure that dynamically adjusted randomisation probabilities to balance key baseline covariates: age, living with children, behavioural intention, BMI, objective physical activity level (ratio of total energy expenditure to estimated basal metabolic rate) [23,26], and gender. Participants visited the measurement centre for over two hours at baseline and 12 months, and completed postal questionnaires at 6 months. For further details about the trial, including the CONSORT diagram and sample size calculation, please refer to Williams et al. (2004) and Kinmonth et al. (2008) [19,20].

### Interventions

All three arms received a theory-based leaflet emphasising the benefits of becoming more physically active and advice to increase physical activity as much as possible.

Participants in the face-to-face and distance arms were additionally offered the intervention program [16]. It was delivered by facilitators from a range of health professions, who received initial training and ongoing supervision. Fidelity of program delivery was promoted through tape-recording of sessions and monthly supervision [17]. The TPB [12] informed the hypothesised mediators of intention and physical activity that were targeted in the intervention program: instrumental and affective attitude, subjective norm and perceived behavioural control. Using the TPB as a theoretical framework, facilitators elicited the participant's beliefs about becoming more physically active: advantages and disadvantages, perceived (lack of) encouragement by important others (e.g., family, friends), and facilitating factors and barriers. Facilitators reinforced positive beliefs and applied problem solving in relation to negative beliefs. Participants were taught a range of self-regulatory strategies to alter cognitions and facilitate behavioural change and maintenance, including goal setting, action planning, self-monitoring, goal review, using rewards, using prompts, building support from family and friends, and relapse prevention. Facilitators encouraged gradual and continuous increases in activity levels. The program lasted one year and began with a home-based introductory session. The face-to-face program included four one-hour home sessions and two 45-minute telephone calls over five months, and monthly follow-up phone calls thereafter. The distance program included six phone calls over five months followed by monthly postal contact.

### Data analysis

Analysis of covariance was used to test intervention impact employing two contrasts: combined interventions (face-to-face and distance) versus brief advice, and face-to-face versus distance. Between-group differences were adjusted for baseline values and stratifiers (age, living with children, behavioural intention, BMI and objective physical activity level balanced across trial arms for gender), and expressed as Cohen's effect size (*d*) using pooled standard deviations. Intervention effects on TPB cognitions were similar when including and excluding behavioural intention as stratifier, and we report effects including this stratifier. Use of the missing indicator method allowed for inclusion in the analyses of participants with missing baseline variables [27]. Outliers were defined as participants with a value at least four standard deviations from the mean, and examined for all outcomes. Outliers were excluded for objectively measured physical activity (*n *= 1 at baseline), self-reported physical activity (*n *= 1 at baseline; *n *= 4 at 12 months), and the belief-based measure of perceived behavioural control (*n *= 1 at 12 months); no outliers were identified at 6 months. Participants with missing follow-up data for individual outcomes were excluded from the respective analysis (see Table 1). Overall missing values were low. Sample size for primary outcome analysis was *n *= 321 (88% of randomised participants; 92% advice, 86% face-to-face, 86% distance; *p *= 0.29 Fisher's Exact test between arms), *n *= 302 for self-reported physical activity at 6 months and *n *= 324 at 12 months. Response rate for the TPB questionnaire was 90% at 6 months and 91% at 12-months; sample sizes for cognitive predictors were based on participants with valid data on the respective construct (see Table 1). Multiple mediation of intervention impact was tested using bootstrapping (5000 bootstrap re-samples) [28], as it has increased power compared to regression analysis, and does not require normal distributions for the estimates of indirect effects (macro on , accessed 11 April 2007). All stratifiers and baseline levels of independent and dependent variables were entered as covariates. Data were analysed in SPSS 12.0.1 and AMOS 7.0.

**Table 1:** Adjusted intervention effect on physical activity and cognitions for combined intervention groups (face-to-face and distance) versus brief advice^a^

	**Combined interventions**			**Brief advice**			**Adjusted intervention effect^c^**			
Outcomes (*n *for *M *(*SD*))	Baseline *M *(*SD*)	6 months *M *(*SD*)^b^	12 months *M *(*SD*)^b^	Baseline *M *(*SD*)	6 months *M *(*SD*)^b^	12 months *M *(*SD*)^b^	6 months Difference in means (95% *CI*)	n	12 months Difference in means (95% *CI*)	n

Objective activity DayPAR (321)^d^	1.84 (.62)	-	1.94 (.65)	1.87 (.55)	-	2.00 (.57)	-		-.04 (-.16 to .08)	321
Self-reported activity (MET hrs/wk) (269)^d^	85.50 (47.80)	99.69 (49.95)	103.95 (59.66)	88.27 (60.30)	97.47 (56.96)	102.71 (63.44)	.51 (-8.78 to 9.80)	302	-.23 (-9.68 to 9.23)	324
Global instrumental attitude (307)	4.47 (.48)	4.38 (.52)	4.34 (.52)	4.46 (.49)	4.29 (.51)	4.28 (.51)	.09 (-.02 to .20)	327	.06 (-.04 to .16)	332
Global affective attitude (308)	3.90 (.57)	3.88 (.62)	3.84 (.58)	3.83 (.66)	3.72 (.64)	3.73 (.64)	.13 (.01 to .26)	329	.09 (-.02 to .21)	332
Global subjective norm (309)	3.63 (.73)	3.54 (.85)	3.57 (.71)	3.49 (.89)	3.38 (.77)	3.41 (.85)	.09 (-.06 to .25)	329	.10 (-.06 to .25)	332
Global perceived behavioural control (309)	3.88 (.58)	3.73 (.69)	3.59 (.72)	3.77 (.62)	3.51 (.69)	3.53 (.70)	.18 (.03 to .32)	329	.05 (-.11 to .20)	332
Behavioural intention (309)	3.72 (.63)	3.85 (.64)	3.65 (.67)	3.71 (.64)	3.53 (.65)	3.57 (.66)	.33 (.20 to .46)	327	.10 (-.04 to .24)	332
Belief-based instrumental attitude (288)	25.46 (10.40)	25.45 (11.38)	25.15 (11.49)	25.89 (10.29)	22.91 (10.67)	22.78 (10.66)	3.78 (1.68 to 5.88)	321	3.61 (1.54 to 5.67)	329
Belief-based affective attitude (308)	8.97 (4.05)	8.63 (4.14)	8.62 (4.02)	8.47 (3.91)	7.81 (4.08)	7.50 (3.78)	.74 (-.06 to 1.55)	328	.96 (.20 to 1.72)	332
Belief-based subjective norm (197)^e^	23.23 (13.28)	21.80 (13.58)	22.21 (12.16)	22.46 (12.22)	20.75 (12.23)	21.63 (11.70)	.89 (-1.96 to 3.75)	214	.47 (-2.17 to 3.11)	223
Belief-based perceived behavioural control (301)^d^	.73 (3.57)	.05 (4.08)	-.73 (4.10)	1.13 (3.90)	-.53 (3.68)	-.26 (3.66)	.66 (-.20 to 1.52)	321	-.04 (-.90 to .83)	331

*Note *^a ^Data on physical activity and intention were reported in Kinmonth et al. [20]. ^b ^Means include participants with baseline missing data. ^c ^Difference in means at 6 and 12 months respectively between combined intervention groups and advice group, adjusted for baseline measure, age, living with children, behavioural intention, BMI and objective physical activity level (PAL) balanced across trial arms for gender. *n *= number of participants on which analysis is based. ^d ^Participants with a value at least four standard deviations from the mean were excluded. ^e ^Only participants who reported having children and a partner at the relevant time-points were included in the analysis.

## Results

### Description of the trial sample

Three-hundred and sixty-five participants were randomised: 120 face-to-face, 124 distance, and 121 brief advice. Sixty-two percent were female and mean (standard deviation) age was 40.4 (6.0). Participants were predominantly white and had finished full-time education at 17.9 (3.2) years; 55.3% had a managerial or professional job. They were overweight (BMI = 27.82 (5.09)) and 19.5% were smokers. Objectively measured physical activity (dayPAR = 1.86 (.62)) indicated that participants were sedentary. They were not worried about developing diabetes (*M*(*SD*) = 9.57 (2.88) on a scale from 6 to 30), and perceived their risk of developing diabetes a little higher than other people of the same age (*M*(*SD*) = 3.72 (.87) on a scale from 1 to 5). Baseline characteristics and randomisation stratifiers were similar across the three trial arms [20].

### Intervention effect on cognitions about increasing physical activity

At baseline, participants were positive about becoming more physically active and had moderately strong intentions. During the year they reported slightly more negative beliefs (Table 1). As there were no significant differences between face-to-face and distance groups at 6 and 12 months, we report differences between intervention groups (face-to-face and distance) and brief advice only.

#### Intervention impact at 6 months (Table 1 and Figure 1)

**Figure 1:**
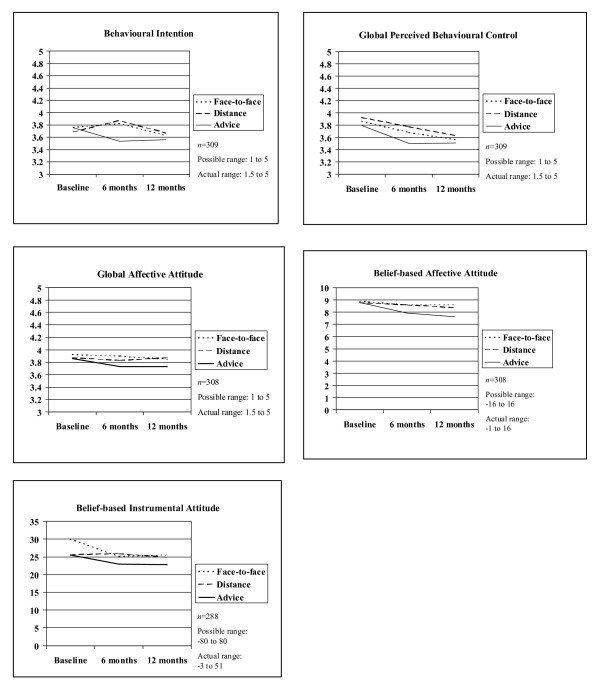
**Mean levels of Theory of Planned Behaviour cognitions among face-to-face, distance and brief advice groups**.

##### Global cognitions

Compared to brief advice, the intervention groups perceived increasing their activity as more enjoyable (adjusted effect (95% CI) on affective attitude .13 (.01 to .26); *d *= .25), and they perceived more control over increasing their activity (.18 (.03 to .32), *d *= .32). Intention became stronger in the intervention groups between baseline and 6 months, but weaker in brief advice (.33 (.20 to .46), *d *= .50). No differences were found in how beneficial participants thought that increasing their activity would be (instrumental attitude) and how much they perceived that important others wanted them to change (subjective norm).

##### Belief-based cognitions

The intervention groups were more positive than the brief advice group about instrumental benefits (e.g., increasing fitness) (3.78 (1.68 to 5.88), *d *= .23). No differences were found for affective attitude, subjective norm and perceived control.

#### Intervention impact at 12 months (Table 1 and Figure 1)

##### Global cognitions

There were no significant differences between intervention groups and brief advice group for any cognitions (all *p*-values > .05).

##### Belief-based cognitions

The intervention groups were more positive than brief advice about instrumental benefits (e.g., preventing diabetes) (3.61 (1.54 to 5.67), *d *= .21) and affective benefits (e.g., feeling better) (.96 (.20 to 1.72), *d *= .29). No differences were found for subjective norm and perceived control.

In sum, intervention impact on cognitions was limited and mostly short-term.

### Do affective attitude and perceived control explain effect on 6-months intention?

**Figure 2:**
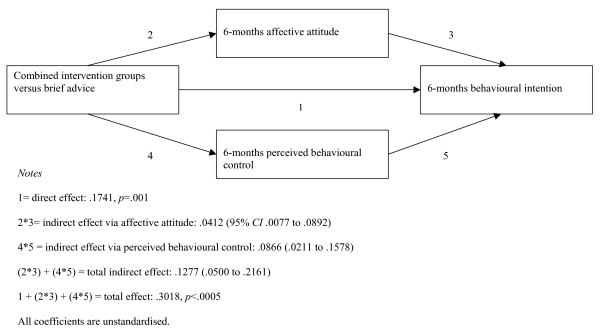
**Multiple mediation of intervention impact (combined interventions versus brief advice) on six-months behavioural intention**.

We conducted multiple mediation analysis to examine whether the effect of the intervention program on 6-months intention was explained by its effect on affective attitude and perceived control (see Figure 2). The two indirect pathways from the intervention program to intention via affective attitude and perceived control, respectively, were significant. The combined indirect pathway was also significant. The direct pathway from the intervention program to intention remained significant after inclusion of the indirect pathways. This is consistent with stronger intentions at 6 months being partially explained by differences in affective attitude and perceived control between the intervention groups and brief advice group.

## Discussion

This paper investigated to what extent an intensive intervention program that failed to increase objective and self-reported physical activity more than brief advice succeeded in changing the hypothesised cognitive predictors of behaviour, and whether any effect on cognitions was consistent with pathways specified by the TPB.

We showed that the effects on cognitions were limited in size, mostly short-term and not sustained at twelve months. A recent meta-analysis suggested that medium to large changes in intention (*d *= .66) may engender small to medium changes in self-reported and objective behaviour (*d *= .36) [29]. The six-months effect on intention in *ProActive *was of this order (*d *= .50), but we found no effect on six-months self-reported behaviour. An objective assessment of physical activity at six months might have given a more precise estimate of effect. In absolute terms, the differences in cognitions between participants receiving the intervention program and those receiving the brief advice leaflet were probably too small (0.1 to 0.2 on a scale of 1 to 5 for most of the global cognitions) to result in behaviour change. Regression-based simulation studies with the TPB applied to 30 behaviours in a student sample [30] and to condom use by adolescents [31] suggest that large intervention effects on many TPB cognitions simultaneously may be needed to produce behaviour change. This suggests that more powerful interventions with large effects on cognitions might have resulted in more physical activity change in our intervention groups. However, evidence from other intervention trials based on a range of theories is mixed: associations between change in theory-based cognitions and increases in physical activity were inconsistent across studies [32-34]. Intervention effects on cognitions in *ProActive *were mainly due to increasingly negative cognitions in the brief advice group over time, rather than more positive cognitions in the intervention groups. This may reflect disappointment about not being offered the intervention program [35]. The intervention groups reported lower perceived control over the year, suggesting that they became more realistic about behaviour change, or could not increase activity any further. With decreasing perceived control, participants may not have used strategies taught to translate intention into action (e.g., action planning).

There are three other potential explanations for the intervention's lack of additional effect on behaviour. First, on average the whole trial cohort showed an increase in activity of 20 minutes of brisk walking per day over the year [20]. This may have been due to the effects of the brief advice leaflet or intensive measurement received by all participants, and may have prevented the intervention program from having an additional effect. Physical activity measurement included a treadmill test and wearing a heart rate monitor, which may have increased awareness of activity levels and facilitated behaviour change without affecting the TPB cognitions measured. Completing TPB questionnaires may have changed behaviour by increasing the saliency of physical activity. In another study the completion of a single TPB questionnaire increased objectively measured blood donation one year later [36].

Second, the intervention program may have been ineffective because we did not include potentially effective behaviour change techniques, such as giving participants specific behavioural targets [37,38]. The intervention program was complex, and interaction between its components may have diluted its effectiveness [39]. The intervention may not have targeted cognitions that are important mediators of physical activity change. While meta-analyses showed that intention and perceived behavioural control are important correlates of self-reported physical activity in community, clinical and student populations [40,41], these cognitions did not predict self-reported and objective physical activity in the *ProActive *cohort (unpublished data).

Finally, insufficient delivery of the intervention program might explain lack of additional impact. Detailed analysis of sessions with 10% of the intervention participants showed that the facilitators applied about half of the behaviour change techniques specified in the protocols [17]. This may have been an insufficient dose to help participants translate their intention into behaviour change.

In this paper we also tested whether any intervention effect on cognitions was consistent with pathways specified by the TPB. We found some support for the theory: stronger intentions among participants of the intervention program were partially explained by affective attitude and perceived control, but not by subjective norm and instrumental attitude. Our study suggests that health practitioners could strengthen patients' intentions to be more active (i) by targeting affective beliefs, for instance through persuasive messages that convey that physical activity is enjoyable, and offering experience of enjoyable activities, and (ii) by targeting perceptions of facilitating factors and barriers, through providing encouragement, modeling the behaviour, or experience in successfully enacting the behaviour [42]. However, our study showed that use of these strategies alone might strengthen intention but not translate into behaviour change. This is consistent with a simulation study in which a substantial minority of students (26%) did not change behaviour in the presence of large effects on several TPB cognitions simultaneously [30]. The gap between intention and action is well-documented [43]. Implementation intentions [44], action planning [45] and strategies to increase actual control over the behaviour [46] are promising strategies to bridge the gap. Action planning partially mediated intervention impact on physical activity in other clinical populations [8,47], and in a meta-analysis social encouragement and incentives for behaving or remaining in the intervention were associated with larger effect sizes on intention and behaviour [29].

Strengths of this study include comprehensive measurement of cognitions targeted in the intervention program and brief advice leaflet, and objective measurement of physical activity. Limitations include lack of a true control group, either for measurement or brief advice, which would have clarified their impact. The poor internal consistency of some of the TPB measures may have resulted in underestimation of the size of mediational pathways.

## Conclusion

The lack of effect of a theory-based intervention program on physical activity over and above brief advice was not due to a complete lack of effect on cognitive predictors, but may be explained by the small, and mostly short-term effects on cognitions. The theoretical basis was supported in that stronger intentions at six months among program participants were partially explained by affective attitude and perceived control. The study raises questions as to whether targeting cognitions based on the TPB is an effective approach to increasing physical activity, and more powerful interventions which induce large changes in TPB cognitions are needed to answer this question. In addition, the results highlight three issues that future research should address. First, studies should investigate a wider range of theory-based mediators of physical activity change, such as those involved in behavioural self-regulation (e.g., behavioural monitoring), unconscious processes (e.g., implicit attitudes), and environmental characteristics. Second, an assessment of participants' use of any self-regulatory strategies (e.g., action planning) taught in the intervention in trial evaluations could enhance our understanding of how people change behaviour. Third, investigators should conduct experimental studies or statistical modeling to test intervention impact on causal pathways between hypothesised mediators and physical activity change, before evaluation in definitive randomised controlled trials. Further evaluations of the impact on behaviour of intensive measurement, and monitoring of physical activity are needed, as well as the effect of simple theory-based advice compared with more complex approaches.

## Competing interests

The authors declare that they have no competing interests.

## Authors' contributions

WH formulated the aims and hypotheses of the study, performed all statistical analyses and drafted the manuscript. SS was the formal PhD supervisor of WH, and contributed to and advised on all the aspects of the study. ALK was principal investigator (PI) of the *ProActive *Trial. SS, ALK and SM contributed to the interpretation of the findings and critical revision of the manuscript. All authors read and approved the final manuscript.
